# An Information Theoretic Approach to Reveal the Formation of Shared Representations

**DOI:** 10.3389/fncom.2020.00001

**Published:** 2020-01-29

**Authors:** Akihiro Eguchi, Takato Horii, Takayuki Nagai, Ryota Kanai, Masafumi Oizumi

**Affiliations:** ^1^Basic Research Group, Araya Inc., Tokyo, Japan; ^2^Department of Systems Innovation, Graduate School of Engineering Science, Osaka University, Osaka, Japan; ^3^International Research Center for Neurointelligence, The University of Tokyo, Tokyo, Japan; ^4^Artificial Intelligence eXploration Research Center, The University of Electro-Communications, Tokyo, Japan; ^5^Department of General Systems Studies, Graduate School of Arts and Science, The University of Tokyo, Tokyo, Japan

**Keywords:** shared representation, auto-encoder, multi-modal data, information theory, mutual information

## Abstract

Modality-invariant categorical representations, i.e., shared representation, is thought to play a key role in learning to categorize multi-modal information. We have investigated how a bimodal autoencoder can form a shared representation in an unsupervised manner with multi-modal data. We explored whether altering the depth of the network and mixing the multi-modal inputs at the input layer affect the development of the shared representations. Based on the activation of units in the hidden layers, we classified them into four different types: visual cells, auditory cells, inconsistent visual and auditory cells, and consistent visual and auditory cells. Our results show that the number and quality of the last type (i.e., shared representation) significantly differ depending on the depth of the network and are enhanced when the network receives mixed inputs as opposed to separate inputs for each modality, as occurs in typical two-stage frameworks. In the present work, we present a way to utilize information theory to understand the abstract representations formed in the hidden layers of the network. We believe that such an information theoretic approach could potentially provide insights into the development of more efficient and cost-effective ways to train neural networks using qualitative measures of the representations that cannot be captured by analyzing only the final outputs of the networks.

## 1. Introduction

The term *concept* describes the fundamental building blocks of thoughts and beliefs we develop in our own mind. Concepts are thought to be crucial for making the predictions required for various tasks in everyday life (Fisher et al., 2014). It has been proposed that concepts are acquired essentially by learning to categorize multi-modal information (Nagai et al., 2016; Nakamura and Nagai, 2017). During this process, modality-invariant categorical representations, i.e., *shared representation*, are thought to be developed. Ngiam et al. (2011) presented a way to develop shared representations in a bimodal autoencoder in an unsupervised manner, and various other researchers have advanced the idea. These activities have highlighted the broad potential utility of multi-modal data, for example in learning sound representations from unlabeled videos (Aytar et al., 2016), learning spoken language with a visual context (Harwath et al., 2016), estimating emotion based on visual and audio cues (Horii et al., 2016), and identifying audio source location in a images (Arandjelovic and Zisserman, 2017a,b), etc.

Nevertheless, these studies have not explicitly investigated the degree to which shared representations can be trained to develop or what aspects are important for the formation of such representations. More specifically, it is still unclear (1) if altering the depth of the encoding layer of an autoencoder and/or (2) mixing the multi-modal data at the input layer facilitates the formation of shared representations.

Previously, it was presented that training a one-layer multi-modal model over the concatenated audio and video data failed to develop shared representations. When the correlations between the multi-modal data are highly non-linear in a “shallow network,” the result is that hidden units that have strong connections to variables from each individual modality (Ngiam et al., 2011). We hypothesized that the quality of shared representation may be better if the network becomes deeper and if the network receives mixed modality data from the input layer.

Based on the activations, we used information theoretic techniques (see section 2.3 for the details) to classify each unit in hidden layers into four different types. The first and second types included cells that represent categories for only a single modality (vision or audio), while the third and fourth types include cells that represent either inconsistent or consistent categories across the two modalities, respectively. We consider that the number of the fourth type indicates the goodness of shared representations.

In order to evaluate the development of shared representations, we also test the actual performance of the network in a context where task performance depends on the successful acquisition and utilization of shared representations. This is achieved by extending the model with additional supervised layers to conduct a “shared-representation learning” (Ngiam et al., 2011). This type of learning would confirm the successful development of shared representations in the encoding layer, and furthermore demonstrate how such representations are useful in solving practical tasks, such as stimulus classification.

Currently, examples of bottlenecks in training deep neural networks (DNNs) include the limited availability of datasets with appropriate annotations and limited strategies to quantitatively evaluate developed representations in intermediate layers (Shwartz-Ziv and Tishby, 2017). As our results show that altering the number of encoding layers and mixing the multi-modal data at the input layer both facilitate the development of shared representations, our information theoretic approach may provide insights into more efficient and cost-effective ways to train various kinds of neural network models with the qualitative measures of the abstract representations developed at the intermediate stages of the networks, which cannot be captured by analyzing only the final outputs of the networks.

## 2. Materials and Methods

### 2.1. Model Description

The current simulation studies were conducted within a bimodal autoencoder developed with the open-source neural network library Keras (Chollet, 2015). This is a variant of a basic autoencoder that consists of the following types of layers arranged in a similar way to the multilayer perceptrons: an input layer, an output layer, and one or more hidden layers. What makes the autoencoder unique is that instead of predicting the target value *Y* given inputs *X*, the model learns to reconstruct its inputs in the output layer by minimizing the difference between the input (*X*) and the output (*X*′) thorough the feature space (*F*) in an unsupervised manner.

More precisely, the same set of data presented at the input serves as a set of teaching signals used at the training within the hourglass-type neural network model where the number of nodes in the hidden layers is smaller than the number of nodes in the input/output layer. As a result, it is expected that an efficient representation for a set of the data will be learned at the hidden layer through data denoising and dimensionality reduction for data visualization (Cottrell and Munro, 1988).

Suppose the number of nodes in the input/output layer is *d* and the number of nodes in the hidden layer is *p*, the input of the encoder *X* = (**x** ∈ ℝ^*d*^), the output of the encoder (the input of the decoder) *F* = (**h** ∈ ℝ^*p*^), and the output of the decoder *X*′ = (**x′** ∈ ℝ^*d*^). Also, when σ and σ′ represent a transfer function, such as a sigmoid function, and *b* represents a bias term, the two phase transitions of encoding and decoding are expressed in the following way:

(1) $$\begin{array}{l} \text{h}=\sigma (\text{Wx}+\text{b})\\ {\text{x}}^{\prime }=\sigma^{\prime }({\text{W}}^{\prime }\text{h}+{\text{b}}^{\prime }) \end{array}$$

During the training, the model aims to minimize reconstruction errors as follows:

(2) $$\begin{array}{l} L(\text{x},{\text{x}}^{\prime })=\|\text{x}-{\text{x}}^{\prime }{\|}^{2}=\|\text{x}-\sigma^{\prime }({\text{W}}^{\prime }(\sigma (\text{Wx}+\text{b}))+{\text{b}}^{\prime }){\|}^{2} \end{array}$$

**Figure 1:**
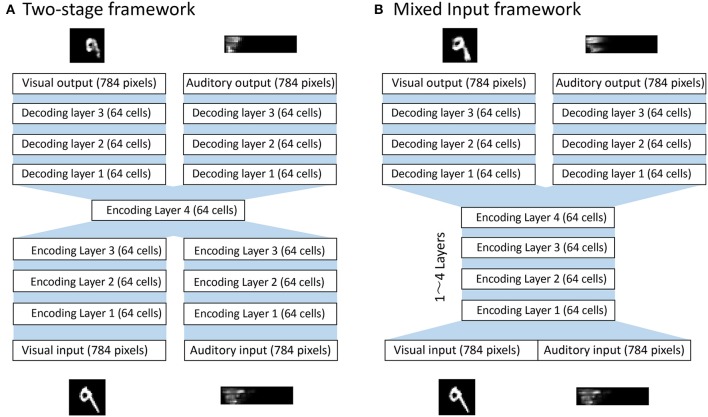
Bimodal deep autoencoder models. **(A)** Two-stage framework that separates feature learning from correlation learning. The network first learns to encode inputs from each modality along 3 consecutive encodings and then learns modality-combined representations at the encoding layer 4. Then, the network learns to reconstruct inputs from each modality along 3 consecutive decoding layers. **(B)** The mixed input framework that concatenates the multi-modal inputs from the beginning. The network first learns to encode the modality-concatenated inputs along encoding layer(s) where the number of layers can vary from 1 to 4 depending on conditions. The network then learns to reconstruct inputs from each modality along 3 consecutive decoding layers. The source code of the simulation model can be downloaded from the github repository at https://github.com/arayabrain/multi-modal-integration.

Figure 1A shows a typical bimodal autoencoder with a two-stage framework to achieve the formation of shared representations, similar to that described in Ngiam et al. (2011). It first learns features separately for each modality and then learns the correlations between those two input modalities. However, a potential weakness of this strategy was subsequently pointed out by Feng et al. (2014) and Peng et al. (2016). These researchers claimed that in reality, two different modalities may be correlated at different abstract levels of representation, and that the two-stage framework may ignore these complex correlations in intermediate representations. We therefore propose instead a model that simultaneously performs the correlation learning and representation learning as a whole, as shown in Figure 1B. With this approach, multi-modal data are mixed from the beginning in the input layer, so the potential risk of ignoring subtle correlations between representations developed at different abstract levels of each modality is minimized.

This model contains two parts to form a bimodal autoencoder: the encoding and the decoding layers. To first encode the multimodal inputs, combined signals of visual and auditory inputs are propagated through a series of encoding layers of 64 cells with sigmoid activation function. Activations in the final encoding layer are then propagated through two parallel paths of multiple layers (from 1 to 4 layers) of 64 cells to reconstruct the signals of each modality. The optimization function used for this model is expressed in the following way, where **x_v_** and ${\text{x}}_{v}^{\prime }$ are the visual input and output while **x_a_** and ${\text{x}}_{a}^{\prime }$ are the audio input and output:

(3) $$\begin{array}{l} L({\text{x}}_{\text{v}},{\text{x}}_{v}^{\prime },{\text{x}}_{\text{a}},{\text{x}}_{a}^{\prime })=(\|{\text{x}}_{\text{v}}-{\text{x}}_{v}^{\prime }{\|}^{2}+\|{\text{x}}_{\text{a}}-{\text{x}}_{a}^{\prime }{\|}^{2}) \end{array}$$

In this particular model, successive neuronal layers are densely connected, and the weights are adjusted via backpropagation of errors with an optimizer of AdaDelta using its default values (Zeiler, 2012) and a loss function of binary cross-entropy during 5,000 epochs of training. After the training, the responses of the cells in the four encoding layers to each pair of stimuli in the testing dataset are used for subsequent analysis. The source code of this simulation model can be found in the github repository at https://github.com/arayabrain/multi-modal-integration.

### 2.2. Dataset and Training Procedure

The visual stimuli used to train and test the network are taken from the database of handwritten digits, *Modified National Institute of Standards and Technology* database (MNIST) (Lecun et al., 1998). Five hundred samples for each digit are taken to construct the training set and 50 samples for each digit are taken to construct the test set. All images are gray-scaled and 28 × 28 pixels in size. The values are rescaled into a range of [0, 1]. Similarly, the auditory stimuli are taken from a publicly available dataset, *free-spoken-digit-dataset* (Jackson, 2018). Datasets for training and testing are generated in the following steps: 100 samples for each digit, which consist of 50 recorded audios of two speakers (namely “Jackson” and “Theo”; Jackson, 2018), are taken to first create a pool of input stimuli. We then randomly select one-half of the stimuli in the dataset for constructing a training set and the second half for use in constructing a test set. Each sound input is transformed into a spectrogram of 14 × 56 pixels in size. The values are rescaled into a range of [0, 1].

Two types of training dataset are created: a dataset consisting of pairs of a visual and an audio input in which the digits from the two modalities correspond with each other (Consistent training dataset), and a dataset consisting of pairs of a visual and an audio input in which the digits do not correspond with each other (Inconsistent training dataset). The inconsistent training dataset is used as a control experiment to evaluate the significance of shared representations developed in the consistent training dataset. In both cases, each of the 500 visual inputs for each digit is paired with a randomly selected input of the 50 auditory inputs. Furthermore, following the procedure used in Ngiam et al. (2011), we use an augmented but noisy dataset with additional examples that have only a single modality as input. In particular, we add examples that contain only zeros for one of the input modalities (e.g., visual inputs) and the original values for the other input modality (e.g., auditory inputs), but require the network to reconstruct both modalities. This means that one-third of the training set has only visual input, another one-third of the set has only auditory input, and the last one-third of the set has both visual and auditory input. Therefore, each training dataset consists of a total of 15,000 pairs of inputs (10 digits × 500 variations × 3 conditions).

In contrast, the test set is created by simply pairing each one of 50 visual inputs for each digit with one of 50 auditory inputs for the corresponding digit. In addition, similarly to the training datasets, we consider those cases where the network is required to reconstruct the signals of two modalities, given that the signals from only one modality are available. Therefore, the dataset is composed of 1,500 pairs of visual and audio inputs (10 digits × 50 variations × 3 conditions).

During the training, the network is exposed to a series of signals coming from visual and auditory modalities assigned in the training set simultaneously, and the weights are adjusted to properly reconstruct both the corresponding visual and auditory signals in the final decoding layers. Once the training is completed, the responses of the cells in each encoding layer of the autoencoder to the input data in the test set are then used for the information analysis described in the next section.

We prepare 10 different consistent and inconsistent training datasets as well as 10 different test datasets according to the above procedures for statistical analysis. We obtain 10 individual results for each of the consistent and inconsistent training.

### 2.3. Information Analysis

In order to analyze the formation of shared representations, we take an information theoretic approach that has traditionally been used in the field of neuroscience. The performance of Deep Neural Networks (DNNs) is typically assessed by the yes/no responses of the units in the output layer, and the activations in the hidden layers tend to be treated as a black box. Recently, however, the use of information theory has gradually gained the attention of AI researchers in various forms (Sorngard, 2014; Berglund et al., 2015; Tishby and Zaslavsky, 2015; Higgins et al., 2016; Shwartz-Ziv and Tishby, 2017; Tax et al., 2017).

In the context of the present study, we are interested in how well the units in the hidden layers of the network have learned to be selective for the digits provided as inputs. Suppose *s* represents the digit that is presented as an input, i.e., s ∈ {0, …, 9}, $\vec{S}$ represents the set of digits presented, and $\vec{R_{i}}$ represents the responses of a particular unit *i* for the set of inputs, the mutual information $I\left(\vec{S},\vec{R_{i}}\right)$ can then provide a single value to summarize the digit selectivity of each unit. However, this measure does not provide information about how selective each cell is for each digit.

In order to identify whether a trained unit is invariantly selective for a particular digit across different modalities, we need to know the amount of information each cell carries about each specific digit. Single cell information analysis described in Rolls et al. (1997) fixes the stimulus *s* and calculates the mutual information $I\left(s,\vec{R_{i}}\right)$ to describe stimulus-specific selectivity.

In this way, if a cell responds invariantly to any inputs of a particular digit but not to inputs of other digits, then the cell carries a high level of information about the presence of its preferred digit (i.e., the cell is maximally selective to the particular digit). From Shannon's definition, we can obtain the expression for the mutual information between the stimulus *s* and the set of responses **R** (the net stimulus information):

(4) $$\begin{array}{l} I(s,{\text{R}}_{\text{i}})=\sum_{r\in {\text{R}}_{\text{i}}}P(r|s)\log_{2}\frac{P(r|s)}{P(r)} \end{array}$$

Here, *P*(*r*|*s*) represents the probability of a specific level of activation of the unit given that a stimulus labeled with a particular digit is presented. *P*(*r*) is estimated on a histogram of values taken by *r* across the presentation of the test set:

(5) $$\begin{array}{l} P\left(r\right)=\underset{s\in \text{S}}{\sum }P\left(r,s\right) \end{array}$$

The maximum information that an ideally developed cell could carry is given by the formula:

(6) $$\begin{array}{l} \text{Maximum cell information }=log_{2}\left(nCat\right)\text{bits} \end{array}$$

where *nCat* is the number of different stimulus categories (the size of **S**).

In our scenario, we consider single-cell information measures for simulation with 10 different digits, from 0 to 9. Therefore, the maximum information possible is *log*_2_(10)≈3.32 bit. To calculate the probability of each response, activity for each cell, *r*, is divided into 10 bins. Using the table of the binned activations, we can measure the information that a particular cell carries about a particular stimulus by calculating the probability of that response *P*(*r*) and the probability of the responses given the stimulus *P*(*r*|*s*) based on the Equation (4).

To provide a solid understanding of the process of computing the amount of the single cell information, let us suppose a simpler scenario with 4 different alphabets, A, B, C, and D (*nCat* = 4), where each alphabet is presented 100 times with different handwriting. Also, for this example, we chose to use only three equally spaced bins, 0 ≤ *r* < 0.33, 0.33 ≤ *r* < 0.67, and 0.67 ≤ *r* ≤ 1. This produces a matrix of responses for each cell, an example is given in Table 1.

**Table 1:** Example cell firing rates to each alphabet over presented in 100 different variations.

**Alphabet**	****0 ≤ *r* < 0.33****	****0.33 ≤ *r* < 0.67****	****0.67 ≤ *r* ≤ 1****	**Total**
A	3	17	80	100
B	68	31	1	100
C	73	25	2	100
D	65	12	23	100
Total	209	85	106	400

Suppose we are interested in the amount of the single cell information that this particular cell carries about an alphabet A. Based on the Equation (4), we first need to calculate the partial information that is specific to each range of the activation in different bins and then to sum each partial information altogether. For example, the strongest range of activation 0.67 ≤ *r* ≤ 1 has the probability of occurring *P*(*r*) = 106/400 and the probability of occurring given that an alphabet A was presented of *P*(*r*|*s*) = 80/100 = 0.8. Therefore, the amount of the information that this particular cell *i* carries about an alphabet A with such a strong activation is *I*(*s, r*) = 0.8log_2_(0.8/(106/400)). We will then need to compute the partial information for the middle range of activation 0.33 ≤ *r* < 0.67 and for the weakest range of activation 0 ≤ *r* < 0.33 in the same manner, and the summation of each partial information gives the final result of the single cell information of this particular cell about an alphabet A, which is in this case about 1.097 bit. Since the single cell information of the same cell about B, C, and D are about 0.380 bit, 0.336 bit, and 0.059 bit, respectively, we can understand that this particular cell carries most information about an alphabet A.

## 3. Results

The main interest of the present study is to understand the nature of concept formation with multi-modal inputs. More specifically, we investigate this process in the context of the formation of *shared representations*, modality invariant categorical representations, in a bimodal auto-encoder. In this section, we provide some of the experimental results to answer the following two questions: (1) Does the quality of the shared representations developed depend on the depth of the network? (2) Does the bimodal autoencoder with the mixed-input framework (see Figure 1) produce better shared representations than that with the two-stage framework?

In particular, we first utilize the information theoretic technique described in methods to quantify the abstract representations that may form in the encoding layers of the bimodal autoencoder and investigate the distribution of the cells with different characteristics. We then conduct the shared-representation learning that aims to test the development of shared representations by evaluating whether inputs from a different modality can be decoded even when only one modality is learned (Ngiam et al., 2011). This is to investigate whether the developed shared representations can actually be utilized to perform digit classification tasks.

### 3.1. Information Analysis

In this section, we first measure how selective each cell in the encoding layers has become to a particular digit presented at each modality after training. Based on the amount of information each cell carries about digits, we identify the number of cells that represent the same digit regardless of the input modality, i.e., the shared representations. We performed simulations 10 times for each condition as described in section 2.2. We first show the results of one simulation to provide the general idea of our information theoretic analysis.

**Figure 2:**
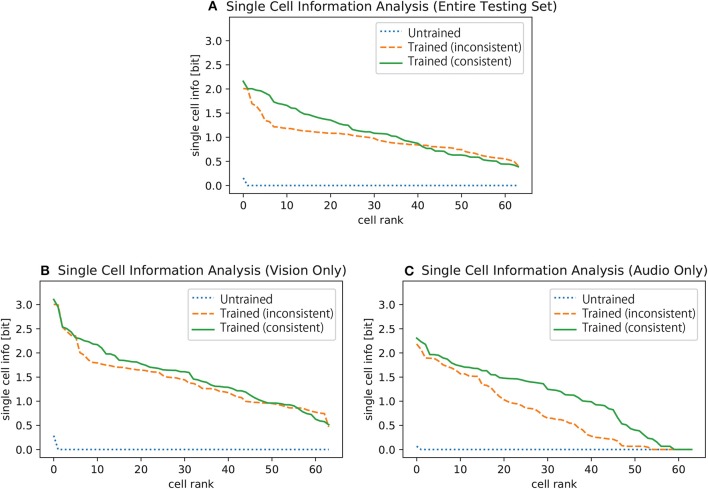
Single cell information analysis of the selectivity of cells to specific digits given **(A)** the entire testing dataset (visual inputs only, audio inputs only, and both visual and auditory inputs), **(B)** visual inputs only, and **(C)** auditory inputs only. All 4th encoding layer cells are plotted along the abscissa in rank order according to the amount of information they convey about the digits. The results before the training are plotted with blue dotted lines, the results of the case where the input digits of both modalities are inconsistent with each other during the training are plotted with orange dashed lines, and the results of the case where the input digits of both modalities are always consistent are plotted with green solid lines.

Figure 2 shows the results of single cell information analysis of the selectivity of cells in the fourth encoding layer of the network to specific digits. Each line plots the maximum amount of single cell information for each cell in the final encoding layer concerning whether one of the ten digits was present. The plot (a) shows the single cell information about the digits given the inputs in the entire test set, which consists of only visual inputs, only auditory inputs, and both visual and auditory inputs (see section 2.2). The results before training are presented with a blue dotted line, which shows that most of the cells carry none of the information about any of the digits at the beginning. On the other hand, the results when the network was trained on the inconsistent training dataset are plotted with an orange dashed line while the results for when the network was trained on the consistent training dataset is plotted with a green solid line. These results show that the networks have learned to carry a higher amount of information about a specific digit after training, and the result for the network trained on the consistent training dataset is generally better than that of the network trained on the inconsistent training dataset.

However, this result does not immediately guarantee that the network has learned to utilize signals from both modalities to represent the digit. For example, let us suppose a cell that responds to any visual presentation of a digit one but not to any auditory presentation of the same digit. In other words, this particular cell responds to only the two-thirds of the subset of the testing dataset that corresponds to the digit one. Nevertheless, the cell can still carry a reasonably high amount of information about the digit one. In order to remove this possibility, the same analysis technique is also applied to the responses of the cells to two different subsets of the testing dataset separately: one-third of the original training dataset which consists of visual inputs only and the dataset which consists of auditory inputs only.

Figures 2B,C shows the results of the single cell information analysis over the subset of the testing dataset that provides signals from only one modality, vision or auditory. We can now confirm with these results that the amount of information carried by each cell concerning a specific digit is higher after training for both modalities.

To understand the nature of the representations in more detail, we classify the cells into four different types, each of which exhibits different selectivity properties in terms of selectivity to visual and audio inputs. (1) Visual cells: selective only to visual inputs. (2) Auditory cells: selective only to auditory inputs. (3) Inconsistent visual and auditory cells: selective to both visual and auditory inputs but selective for different digits. (4) Consistent visual and auditory cells: selective to both visual and auditory inputs and selective for at least one same digit. The existence of type (4) cells indicates to what extent shared representations are developed during the learning process. We classify the cells as “selective” if the amount of information exceeds a certain threshold value. We set the threshold to 0.96 bits for visual inputs and 0.94 bits for auditory inputs, respectively. This threshold value is determined based on the 80th percentile of the amount of the information each cell carries about each digit of the corresponding modality in the fourth encoding layer of the network after training on the consistent training dataset.

**Figure 3:**
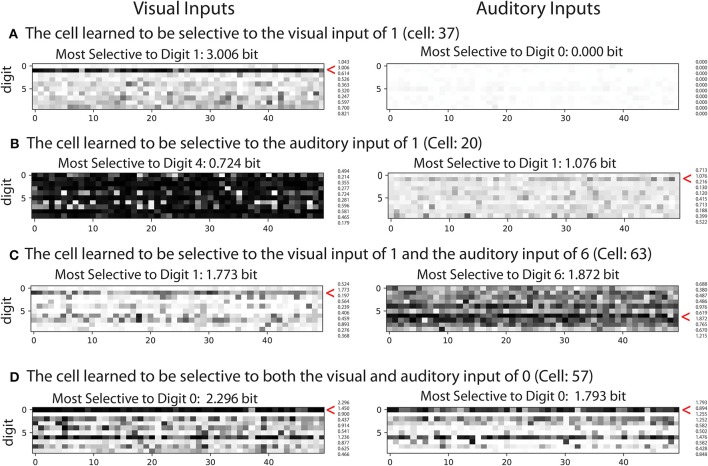
Examples of cell activations. Each row shows the activations of different example cells. The plot shows the responses of the cells to 50 variations of visual input (left) and auditory input (right) for each digit. The darker the color is, the higher the activation. **(A)** Visual cell: an example of a cell that learned to be selective to digit one of the visual input. **(B)** Auditory cell: an example of a cell that learned to be selective to digit one of the auditory input. **(C)** Inconsistent visual and auditory cell: an example of a cell that represents inconsistent digits across the modalities. **(D)** Consistent visual and auditory cells (shared representation): an example of a cell that represents the same digits.

Figure 3 shows the activation of exemplar cells of the four types defined above. For more details of the amount of information that each cell carries about different visual and auditory inputs, please refer to Table S1. The plot in the first column shows the response of the cells to 50 variations in visual input for each digit, and the plot in the second column shows those of auditory input. (a) shows a cell that carries 3.006 bits of information about digit “1” from the visual input but no information from the auditory input. (b) shows a cell that carries 1.076 bits of information about digit “1” from auditory input and not from visual input (the maximum information is 0.724 bit about digit 4). (c) shows a cell that learned to represent digit “1” from the visual input and digit “6” from the auditory input (inconsistent digit depending on modality). (d) shows the example of a cell that learned to respond to digit “0” regardless of the modality the signal comes from, which can be regarded as the shared representation. Note that the most active cell does not necessarily mean the most selective cell, but this figure shows the cases in which the cells carry high mutual information and are also highly active for the sake of better visualization.

**Figure 4:**
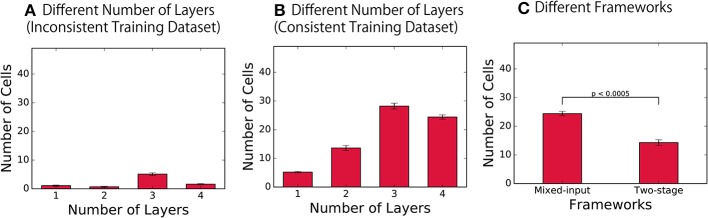
Distribution of the cells with different selectivity properties. **(A,B)** Number of consistent visual and audio cells (shared representation) in the final layer of the network with different depths, trained on the inconsistent training dataset and on the consistent training dataset, respectively. **(C)** Results of the networks with the two different architectures.

In order to understand the development of such representations, the number of hidden encoding layers was varied, and the two types of the network architecture, i.e., mixed-input and two-stage framework, were compared, as described in section 2.1. Based on the information calculated for each modality, we quantified the distribution of the four types of cells that learned to exhibit the different selectivity properties. Figure 4 shows the average number of consistent visual and auditory cells (regarded as shared representation) over 10 simulations. The distribution of three other types of cells are presented in the supplementary material (Figures S1–S7, Tables S2–S6).

As a control experiment, we first trained the network with inconsistent training dataset, in which different visual and auditory inputs are paired. The result presented in Figure 4A confirms that the number of consistent visual and auditory cells developed was very small, which indicates the failure of formation of shared representations in the control condition. On the other hand, when the same network was trained on the consistent training dataset, the network successfully developed a significantly larger number of such cell types, as shown in Figure 4B (*t*-test, *p* < 0.0005 in each layer). By using the results obtained with inconsistent training datasets as baseline, this result confirms the successful development of shared representations across all mixed-input networks, each implemented with a different depth.

More importantly, these results revealed the fact that the number of units with shared representations significantly changes as the depth of the network alters [one-way ANOVA, *F*_(3, 36)_ = 156.24, *p* < 0.0005]. Ngiam et al. (2011) has previously presented that the number of the units with shared representations formed in a shallow network is limited, which is consistent with our present results. However, while these experiments propose the workaround of introducing the two-stage framework, our results show that the problem is not necessarily due to a limitation of the original mixed-input framework; rather, simply increasing the number of layers in the network implemented with the mixed-input framework may also be a simple workaround.

To investigate the difference in the formation of shared presentations between these two different network architectures, we implemented a network with two-stage framework as described in section 2.1. The quality of the representations formed in the final encoding layer of the model was compared with that of representations formed in our model with the mixed-input framework in Figure 4C. The results indicate the fact that the number of consistent visual and auditory cells (i.e., shared representations) is significantly larger in the model implemented with the mixed-input framework (*t*-test, *p* < 0.0005). This leads to the conclusion that the shared representations are better achieved in the mixed-input than the two-stage framework.

### 3.2. Shared Representation Learning

In this section, we test the development of shared representations by evaluating whether digits from different modalities can be decoded even when only one modality is learned. We conducted this test by implementing an additional supervised layer for learning to decode the digits. In particular, we conducted a test for “shared representation learning” to evaluate if the categorical representations developed in the final encoding layer of the bimodal autoencoder capture correlations across different modalities. This test additionally allows us to assess whether the learned representations are modality-invariant and exhibit the characteristics of the shared representations based on a digit classification task.

**Figure 5:**
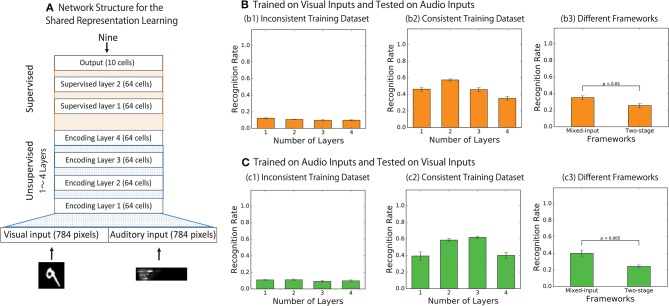
**(A)** The network structures for shared representation learning. The final encoding layer of the bimodal autoencoder is treated as an input layer for the following additional hierarchical neural network to achieve digit classification via supervised learning. **(B,C)** Results of the shared representation learning. Categorical accuracies of the output responses where the network was trained with supervised signals over the visual inputs only **(B)** and over the audio inputs only **(C)** are presented. **(B1,C1)** Show the results when the network was trained on the inconsistent training dataset while **(B2,C2)** Show the results when the network was trained on the consistent training dataset, and **(B3,C3)** compare the performance of the network implemented with the two different frameworks.

Figure 5A illustrates the modified architecture of the network used to conduct the test of the shared representation learning (Ngiam et al., 2011). In particular, the final encoding layer of the bimodal autoencoder described in Figure 1B is treated as an input layer for the following additional neural network layers to achieve a digit classification via supervised learning. The supervised layer consists of three densely connected neuronal layers: two layers of 64 rectified linear units with 20% dropout (Hinton et al., 2012) and a layer of 10 cells with softmax activation function to represent each digit.

During the shared representation learning, the weights of the bimodal autoencoder are fixed while the weights of the additional supervised layers are adjusted to identify the digit of the incoming signals. To test the modality invariance learning, the network is trained on only one modality (e.g., vision) and is then tested on another modality (e.g., auditory), on which the network has never been explicitly trained. If the network has successfully developed the shared representation, it is expected that the categorical accuracy of digit prediction based on signals from this never-trained modality would also be improved. In order to assess the statistical significance of the results, we conducted the training 10 times for each condition.

Figures 5B,C shows the results of the shared representation learning. The figure plots the average categorical accuracy of the responses of the trained output cells over 10 simulations. Figure 5B shows the results when the labels are trained with the visual inputs while Figure 5C shows the results when the labels are trained with the auditory inputs. For each condition, the results are compared between the cases in which the bimodal autoencoder has been trained on the inconsistent training set (Figures 5B1,C1) and on the consistent training set (Figures 5B2,C2). The dashed blue lines represent the results of testing with visual inputs, and the solid orange lines represent the results of testing with auditory inputs.

The result presented in Figures 5B,C confirms that the bimodal autoencoder trained on the inconsistent training dataset failed to develop the shared representations whereas the network trained on the consistent training dataset did successfully develop the shared representations.

Also, we compared the results between the autoencoders implemented with the two-stage framework and the mixed-input framework. In the model implemented with the two-stage framework, the final encoding layer of each network is used as the input to train the following supervised layers to achieve the shared representation learning. Figures 5B3,C3 shows the result after training with the visual inputs and with the auditory inputs, respectively. In both cases, the effect of shared representation learning within the network implemented with the mixed-input framework is better than that in the network implemented with the two-stage framework (*t*-test, *p* < 0.05 in Figure 5B3 and *t*-test, *p* < 0.005 in Figure 5C3. This finding is consistent with the results reported in section 3.1.

For reference, the complete set of results with all the different conditions tested is presented in Figures S8–S14, Tables S7–S11.

## 4. Discussion

In this study, we revisited the development of a specific internal representation emerging in a neural network model originally investigated in Ngiam et al. (2011). Together with this investigation, we also aimed to establish a technique to quantitatively measure the representations in the different layers during the training to understand the black box. More precisely, we utilized an information theoretic approach that has traditionally been used in the field of neuroscience (Rolls et al., 1997), further refined by Eguchi et al. (2016). The use of information theory provided a means of measuring the quality of representations formed in the hidden layers based on the response patterns of the cells. With this technique, we confirmed that the emergence of modality-invariant categorical representations (i.e., shared representations) could be directly assessed even at an abstract level, and thereby successfully described how the network may make use of multi-modal data to develop such representations.

In particular, we investigated the effect of changing the depth of the network and the effect of implementing different frameworks on the formation of shared representations. We confirmed that the network can develop shared representations in a simple bimodal autoencoder (Figures 2, 3) and found that the proportion of cells with shared representations significantly changed depending on the depth of the network (Figure 4B). We also highlighted the potential of using a mixed-input framework rather than the typical two-stage framework by presenting the larger number of shared representations developed in the former (Figure 4C) and the better performance of shared representation learning (Figures 5B3,C3).

As shown in the present study, information theoretic assessment provided a way to quantitatively and qualitatively understand the various kinds of representations emerging in the models. Our approach clarified the effect of model structure (i.e., depth and mixed-method of multimodal signals) in the acquisition of categorical representations and the relationship between shared representations and input signals. This approach might help to evaluate previous studies. For example, some previous studies Horii et al. (2016, 2018) used a two-stage framework to acquire emotional categories from human visual and auditory signals. However, these did not evaluate performance when the model structures were changed. Thus, it is possible that the mixed-input framework provides better performance in shared representation learning, as indeed we showed in this study.

With regard to the effectiveness of using mutual information to characterize the representations of hidden units, some of the recent attempts (Chen et al., 2016; Hjelm et al., 2018; Pineau and Lelarge, 2018; Amjad and Geiger, 2019; Zhao et al., 2019) directly introduced mutual information in the objective function. It has been shown that those novel learning rules help to acquire and improve the disentangled representation in the hidden layers. We expect that the method proposed in this study could potentially open up the black box of the deep neural network and provide insights into the development of more efficient and cost-effective ways to train the networks.

## Data Availability Statement

Publicly available datasets were analyzed in this study. This data can be found here: http://yann.lecun.com/exdb/mnist/.

## Author Contributions

AE and TH performed the research. AE, TH, and MO wrote the paper. All authors designed the research, discussed the results, and reviewed the final manuscript.

### Conflict of Interest

AE, RK, and MO were employed by company Araya Inc. The remaining authors declare that the research was conducted in the absence of any commercial or financial relationships that could be construed as a potential conflict of interest.
