# Supplementary material for: An Information Theoretic Approach to Reveal the Formation of Shared Representations
Source: Front Comput Neurosci. 2020 Jan 29;14:1. doi: 10.3389/fncom.2020.00001 (PMC7001587; doi:10.3389/fncom.2020.00001)
Supplement: Supplementary file 1 [file Data_Sheet_1.PDF]

Supplementary Material

1 TABLES

Table S1. Information of each cell about different visual and auditory inputs presented in Figure 3

digit	(a) Visual Cell		(b) Auditory cell		(c) Inconsistent visual and auditory cell		(d) Consistent visual and auditory cells	
	Visual Inputs	Auditory Inputs	Visual Inputs	Auditory Inputs	Visual Inputs	Auditory Inputs	Visual Inputs	Auditory Inputs
0	1.043	0.000	0.494	0.713	0.524	0.688	2.296	1.793
1	3.006	0.000	0.214	1.076	1.773	0.380	1.450	0.894
2	0.614	0.000	0.355	0.216	0.197	0.487	0.900	1.255
3	0.526	0.000	0.277	0.130	0.564	0.486	0.437	1.252
4	0.363	0.000	0.724	0.120	0.239	0.976	0.914	0.582
5	0.320	0.000	0.281	0.415	0.406	0.619	0.541	0.502
6	0.247	0.000	0.596	0.713	0.459	1.872	1.236	1.476
7	0.597	0.000	0.581	0.188	0.893	0.765	0.877	0.582
8	0.700	0.000	0.465	0.399	0.276	0.670	0.625	0.428
9	0.821	0.000	0.179	0.522	0.368	1.215	0.466	0.848

Table S2. Comparison between the results after training with *consistent training dataset* and *inconsistent training dataset* (1 layer model) (Figure S3).

	Consistent Training	Inconsistent Training	t value	p value
Visual Cell	5.400 ± 0.514	5.800 ± 0.613	-0.474	0.641
Auditory cell	19.30 ± 1.180	23.00 ± 0.980	2.290	0.034
Inconsistent visual and auditory cells	4.800 ± 0.395	6.300 ± 0.617	-1.940	0.071
Consistent visual and auditory cells	5.200 ± 0.237	1.100 ± 0.221	12.00	5.300×10^{-10}

Table S3. Comparison between the results after training with *consistent training dataset* and *inconsistent training dataset* (2 layer model) (Figure S4).

	Consistent Training	Inconsistent Training	t value	p value
Visual Cell	5.000 ± 0.548	20.30 ± 1.120	-11.60	2.950×10^{-8}
Auditory cell	24.10 ± 1.080	16.50 ± 0.587	5.860	4.290×10^{-5}
Inconsistent visual and auditory cells	11.00 ± 0.927	7.800 ± 1.160	2.040	0.057
Consistent visual and auditory cells	13.60 ± 0.908	0.700 ± 0.202	13.20	1.360×10^{-7}

Table S4. Comparison between the results after training with *consistent training dataset* and *inconsistent training dataset* (3 layer model) (Figure S5).

	Consistent Training	Inconsistent Training	t value	p value
Visual Cell	11.70 ± 0.664	24.70 ± 1.060	-9.870	5.500×10^{-8}
Auditory cell	6.800 ± 0.465	10.90 ± 0.888	-3.880	0.002
Inconsistent visual and auditory cells	14.50 ± 0.587	19.00 ± 0.529	-5.400	4.090×10^{-5}
Consistent visual and auditory cells	28.20 ± 1.030	5.100 ± 0.457	19.50	1.090×10^{-10}

Table S5. Comparison between the results after training with *consistent training dataset* and *inconsistent training dataset* (4 layer model) (Figure S6).

	Consistent Training	Inconsistent Training	t value	p value
Visual Cell	17.40 ± 0.696	35.80 ± 1.260	-12.20	7.560×10^{-9}
Auditory cell	4.200 ± 0.395	8.100 ± 0.607	-5.110	1.180×10^{-4}
Inconsistent visual and auditory cells	10.80 ± 0.785	9.000 ± 1.520	0.997	0.337
Consistent visual and auditory cells	24.40 ± 0.764	1.600 ± 0.210	27.30	5.570×10^{-11}

Table S6. Comparison between the results with Mixed-input model and with two-stage model after training with *consistent training dataset* (Figure S7).

	Mixed-input	Tow-stage	t value	p value
Visual Cell	17.40 ± 0.696	23.00 ± 1.210	-3.810	0.002
Auditory cell	4.200 ± 0.395	7.000 ± 0.616	-3.630	0.002
Inconsistent visual and auditory cells	10.80 ± 0.785	12.80 ± 0.810	-1.680	0.110
Consistent visual and auditory cells	24.40 ± 0.764	14.30 ± 0.990	7.660	6.790×10^{-7}

Table S7. Comparison between the categorical accuracies of the output responses after the shared representation learning with the input 1 layer network trained with consistent training dataset and with the input cells trained with inconsistent training dataset (Figure S10).

	Consistent Training	Inconsistent Training	t value	p value
Visual to Visual	0.932 ± 0.004	0.939 ± 0.004	-1.070	7.160×10^{-8}
Visual to Audio	0.463 ± 0.024	0.122 ± 0.007	12.90	0.034
Audio to Visual	0.394 ± 0.051	0.109 ± 0.007	5.270	4.620×10^{-4}
Audio to Audi	0.727 ± 0.041	0.748 ± 0.008	-2.330	0.035

Table S8. Comparison between the categorical accuracies of the output responses after the shared representation learning with the input 2 layer network trained with consistent training dataset and with the input cells trained with inconsistent training dataset (Figure S11).

	Consistent Training	Inconsistent Training	t value	p value
Visual to Visual	0.923 ± 0.003	0.937 ± 0.004	-2.680	0.016
Visual to Audio	0.575 ± 0.013	0.107 ± 0.003	32.50	2.170×10^{-11}
Audio to Visual	0.586 ± 0.020	0.110 ± 0.009	20.10	5.450×10^{-11}
Audio to Audi	0.728 ± 0.009	0.707 ± 0.008	1.600	0.128

Table S9. Comparison between the categorical accuracies of the output responses after the shared representation learning with the input 3 layer network trained with consistent training dataset and with the input cells trained with inconsistent training dataset (Figure S12).

	Consistent Training	Inconsistent Training	t value	p value
Visual to Visual	0.922 ± 0.005	0.929 ± 0.005	-0.985	0.338
Visual to Audio	0.457 ± 0.024	0.097 ± 0.010	13.10	2.410×10^{-8}
Audio to Visual	0.618 ± 0.013	0.091 ± 0.014	25.80	1.370×10^{-15}
Audio to Audi	0.707 ± 0.005	0.688 ± 0.007	2.180	0.044

Table S10. Comparison between the categorical accuracies of the output responses after the shared representation learning with the input 4 layer network trained with consistent training dataset and with the input cells trained with inconsistent training dataset (Figure S13).

	Consistent Training	Inconsistent Training	t value	p value
Visual to Visual	0.918 ± 0.005	0.940 ± 0.006	-2.750	0.013
Visual to Audio	0.351 ± 0.023	0.098 ± 0.007	10.10	9.280×10^{-7}
Audio to Visual	0.401 ± 0.037	0.098 ± 0.012	7.480	1.270×10^{-5}
Audio to Audi	0.660 ± 0.012	0.666 ± 0.010	-0.366	0.718

Table S11. Comparison between the categorical accuracies of the output responses after the shared representation learning with the input network (4 layers) trained with consistent training dataset with the mixed-input model and with the two-layer network model (Figure S14).

	Mixed-input	Tow-stage	t value	p value
Visual to Visual	0.918 $\pm$ 0.005	0.932 $\pm$ 0.004	-2.080	0.053
Visual to Audio	0.351 $\pm$ 0.023	0.255 $\pm$ 0.025	2.700	0.015
Audio to Visual	0.401 $\pm$ 0.037	0.242 $\pm$ 0.019	3.650	0.003
Audio to Audi	0.660 $\pm$ 0.012	0.738 $\pm$ 0.007	-5.470	8.070×10^{-5}

2 FIGURES

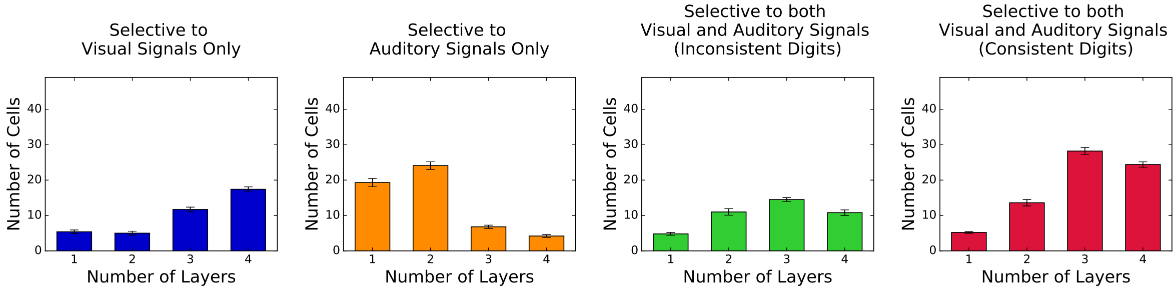

Figure S1. Distribution of the cells with different selectivity properties developed after training with the *consistent training dataset*.

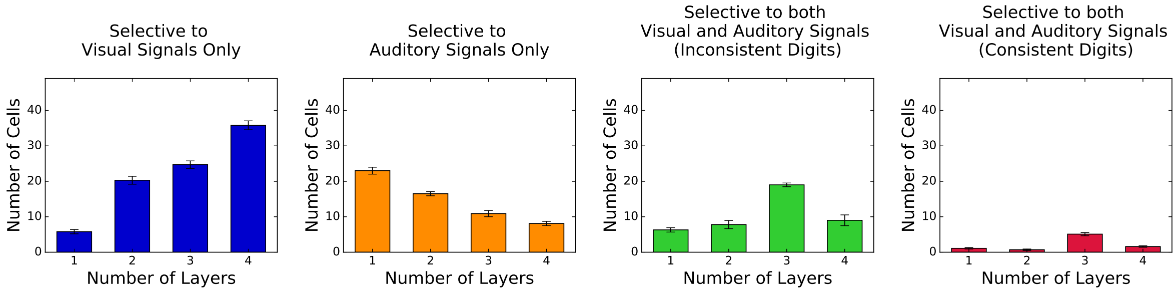

Figure S2. Distribution of the cells with different selectivity properties developed after training with the *inconsistent training dataset*.

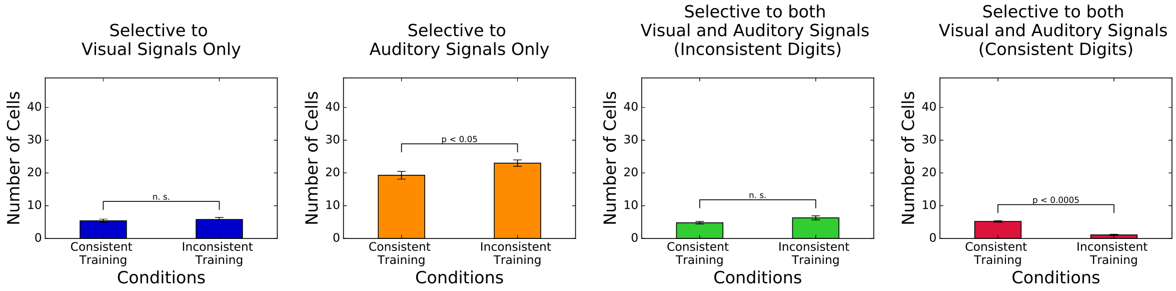

Figure S3. Comparison between the results after training with *consistent training dataset* and *inconsistent training dataset* (1 layer model).

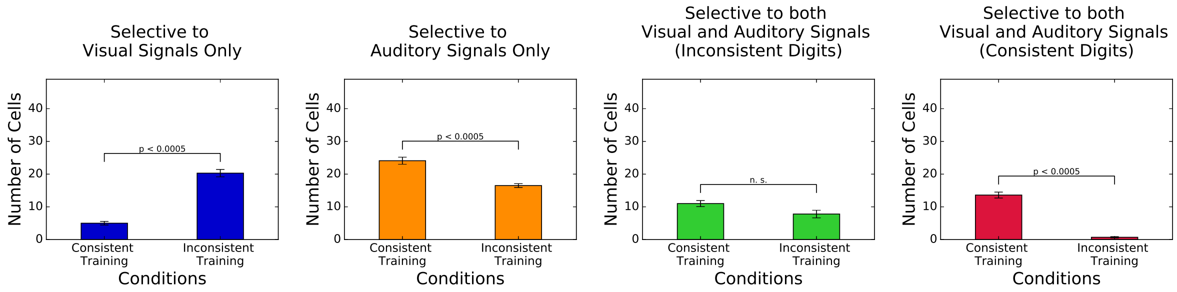

Figure S4. Comparison between the results after training with *consistent training dataset* and *inconsistent training dataset* (2 layer model).

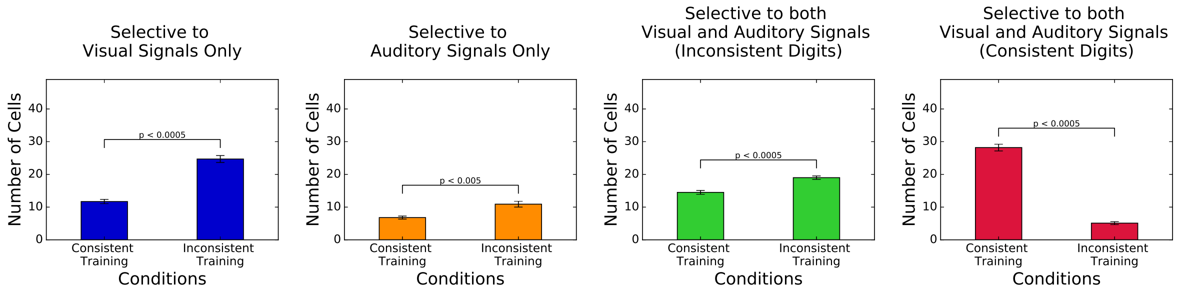

Figure S5. Comparison between the results after training with *consistent training dataset* and *inconsistent training dataset* (3 layer model).

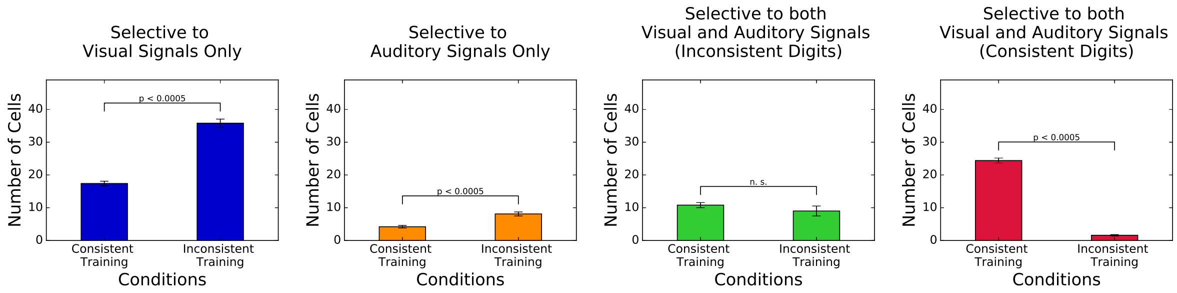

Figure S6. Comparison between the results after training with *consistent training dataset* and *inconsistent training dataset* (4 layer model).

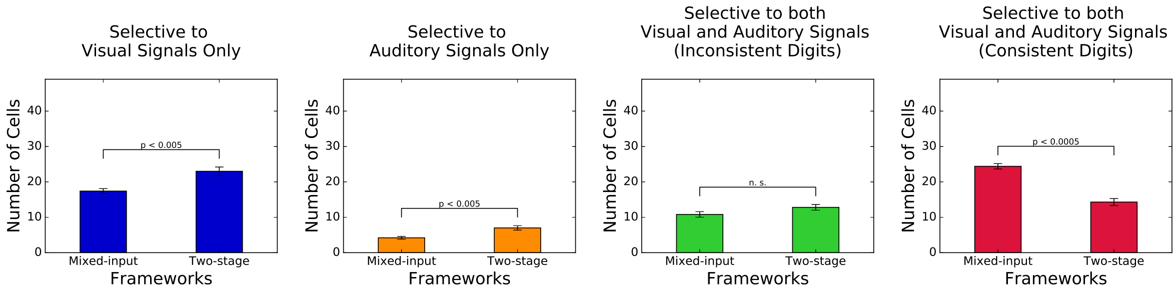

Figure S7. Comparison between the results with Mixed-input model and with two-stage model after training with *consistent training dataset*.

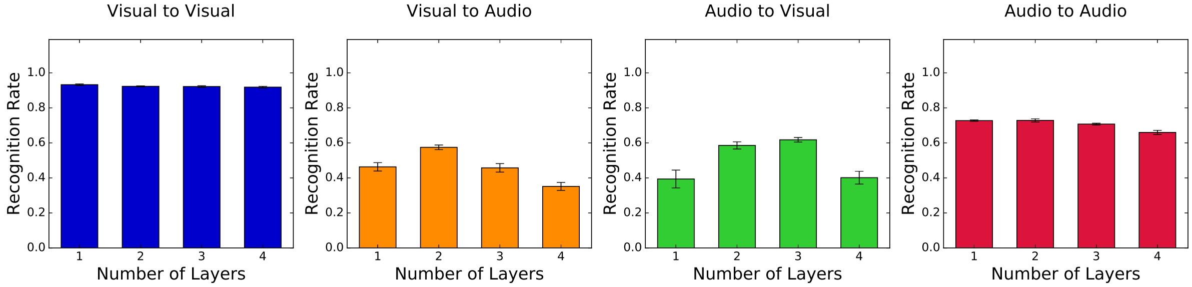

Figure S8. Categorical accuracies of the output responses after the shared representation learning with the input cells trained with consistent training dataset.

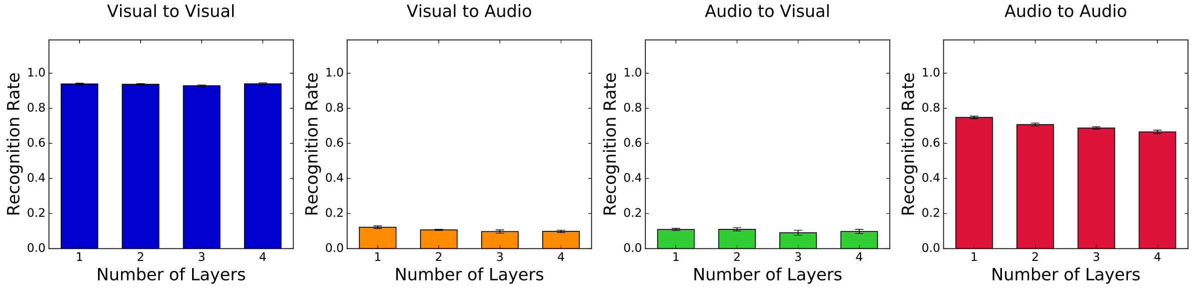

Figure S9. Categorical accuracies of the output responses after the shared representation learning with the input network trained with inconsistent training dataset.

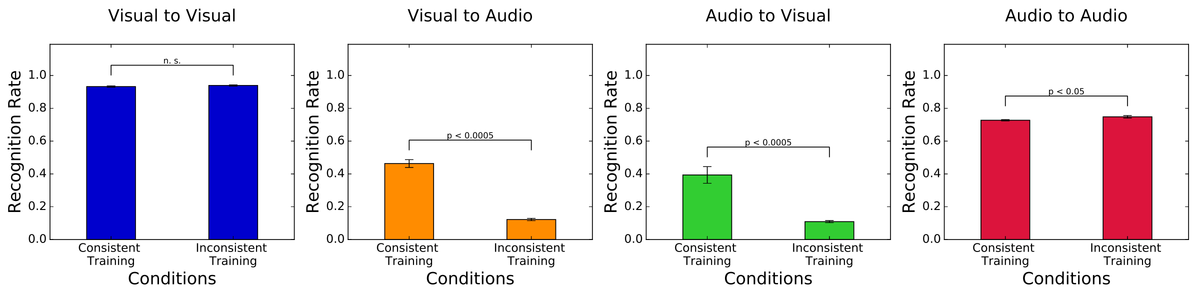

Figure S10. Comparison between the categorical accuracies of the output responses after the shared representation learning with the input 1 layer network trained with consistent training dataset and with the input cells trained with inconsistent training dataset.

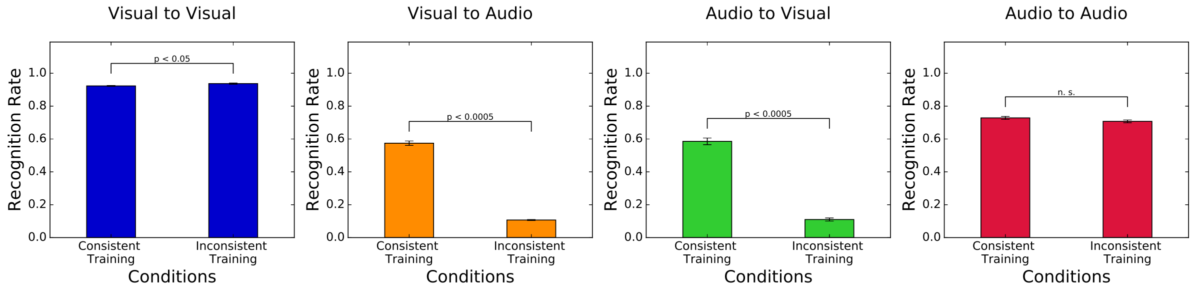

Figure S11. Comparison between the categorical accuracies of the output responses after the shared representation learning with the input 2 layer network trained with consistent training dataset and with the input cells trained with inconsistent training dataset.

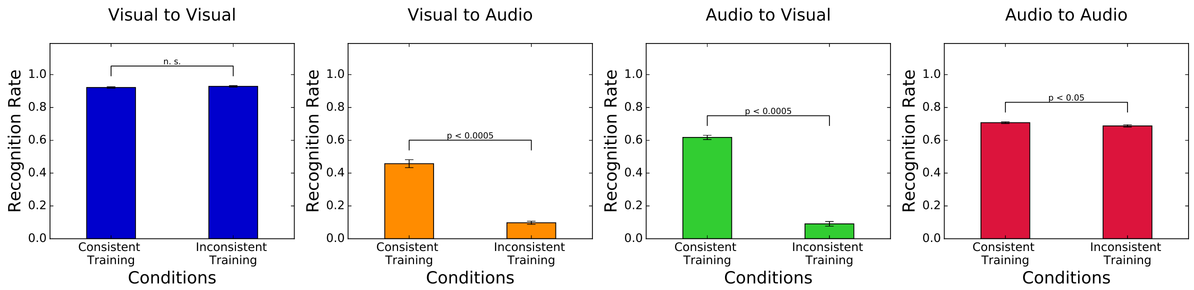

Figure S12. Comparison between the categorical accuracies of the output responses after the shared representation learning with the input 3 layer network trained with consistent training dataset and with the input cells trained with inconsistent training dataset.

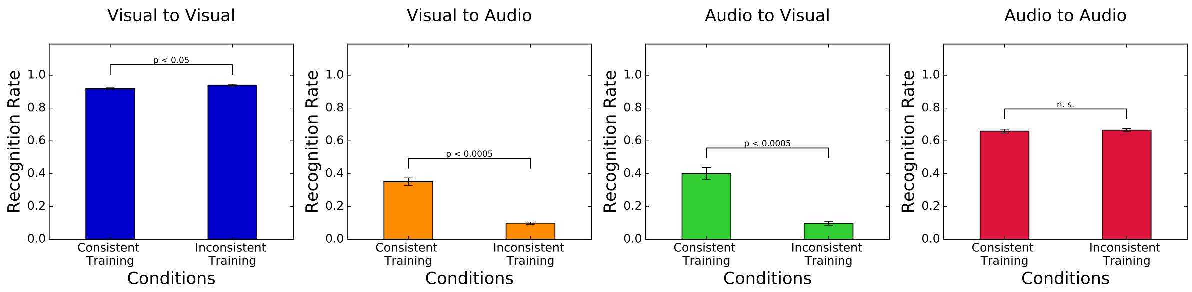

Figure S13. Comparison between the categorical accuracies of the output responses after the shared representation learning with the input network (4 layers) trained with consistent training dataset and with the input cells trained with inconsistent training dataset.

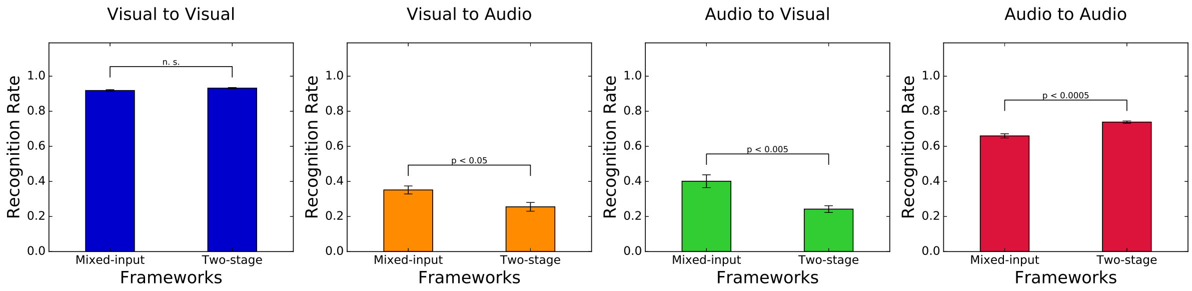

Figure S14. Comparison between the categorical accuracies of the output responses after the shared representation learning with the input network (4 layers) trained with consistent training dataset with the mixed-input model and with the two-layer network model.
